# Rapid Resolution of Acute Fulminant Myocarditis after IVIG and Steroid Treatment

**DOI:** 10.1155/2012/262815

**Published:** 2012-12-04

**Authors:** Michael Barrie, Lucas McKnight, Pallavi Solanki

**Affiliations:** ^1^Department of Emergency Medicine, The Ohio State University Wexner Medical Center, Columbus, OH 43210, USA; ^2^Department of Internal Medicine and Department of Pediatrics, The Ohio State University Wexner Medical Center, Columbus, OH 43210, USA; ^3^Division of Cardiovascular Medicine, Department of Internal Medicine and The Dorothy M. Davis Heart and Lung Research Institute, The Ohio State University Wexner Medical Center, Columbus, OH 43210, USA

## Abstract

We report a case of a 59-year-old woman who presented with worsening dyspnea which rapidly progressed to severe heart failure. Coronary arteries showed no obstruction. Supportive measures stabilized the patient's hemodynamics. Initially intravenous solumedrol was given, but when the patient's condition continued to deteriorate, intravenous immunoglobulin (IVIG) was added to the treatment regimen and her condition improved. Studies show no benefit to using immunosuppressive agents in viral myocarditis, but benefits have been demonstrated in other etiologies. Patients presenting with acute fulminant myocarditis with unknown etiology that continue to deteriorate with aggressive heart failure treatment may benefit from steroids and IVIG.

## 1. Introduction

Myocarditis describes a group of heterogeneous disease processes that all involve inflammation of the myocardium. Diagnosis can be challenging due to nonspecific symptoms. Initiating causes include infectious causes and noninfectious etiologies such as toxins or primary immunologic processes. Acute myocarditis refers to the initial insult where pathogens or toxins invade the myocardium and directly cause myocyte damage. The innate immune response to the insult can release previously hidden autoantigens, which then trigger a subacute autoimmune responses in susceptible individuals [1]. This immunologic understanding has led to the use of immunosuppressive therapies, including steroids and intravenous immunoglobulin (IVIG), to treat fulminant myocarditis in addition to supportive heart failure treatment [2]. However, the efficacy of IVIG and steroids to prevent long-term systolic dysfunction remains controversial. We report a case of a middle-aged female with acute fulminant myocarditis who recovered rapidly after treatment with IVIG and steroids.

## 2. Case Report

**Figure 1:**
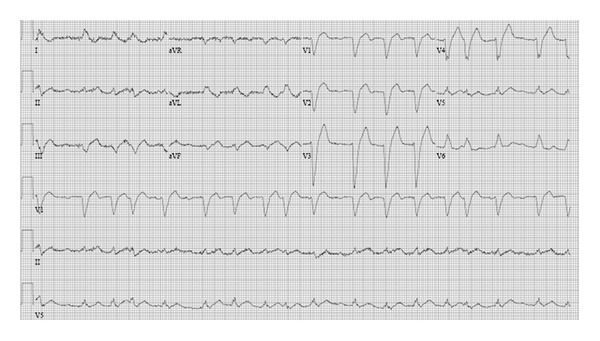
ECG on presentation demonstrating new onset atrial fibrillation and LBBB.

A 59-year-old woman with no prior cardiac history presented in the springtime to an outside hospital with one month of progressive fatigue, palpitations, and exertional chest pain. Past medical history included chronic lumbar and cervical disc disease with cervical vertebral body fixation ten years previously, with continued chronic pain. Current medications were unknown at presentation. She was found to have elevated cardiac enzymes, a new left bundle branch block (LBBB), and new onset atrial fibrillation on electrocardiogram (ECG) (Figure 1). 

These findings prompted evaluation with a left heart catheterization and coronary angiography to evaluate for acute coronary syndrome. Angiography showed nonobstructive coronary artery disease. Transthoracic echocardiogram showed biventricular heart failure with global hypokinesis with left ventricular ejection fraction (LVEF) of 30%. She was treated for suspected myopericarditis with nonsteroidal anti-inflammatory drugs (NSAIDs) and diuresis. Atrial fibrillation was treated with a loading dose of intravenous amiodarone. However, she continued to decompensate and developed respiratory distress and hypotension refractory to vasopressors. 

**Figure 2:**
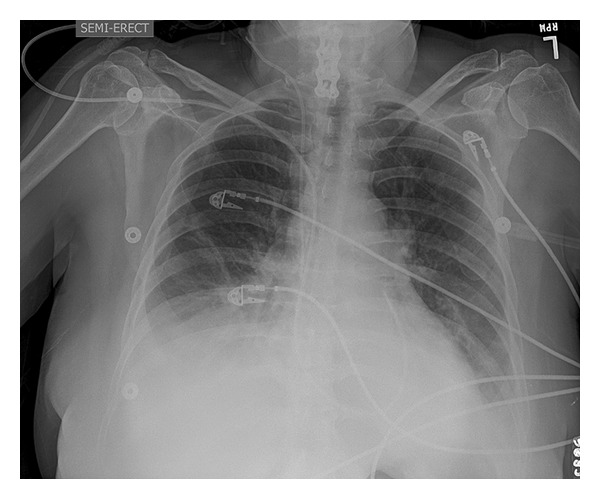
Chest plain film on presentation demonstrating likely pulmonary edema without cardiomegaly.

**Figure 3:**
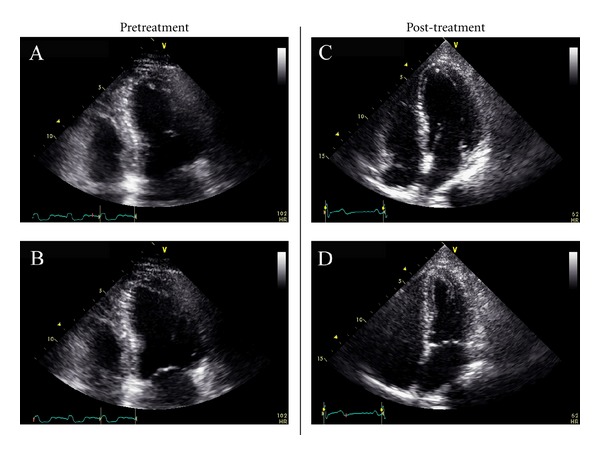
Transthoracic echocardiogram before and after treatment with IVIG and steroids. Apical 4-chamber view with pretreatment end diastole (A) and end systole (B) demonstrating severe global systolic dysfunction, LVEF <20%. The repeat transthoracic echocardiogram before treatment prior to discharge end diastole (C) and end systole (D) demonstrating a near complete resolution of ventricular systolic dysfunction.

**Figure 4:**
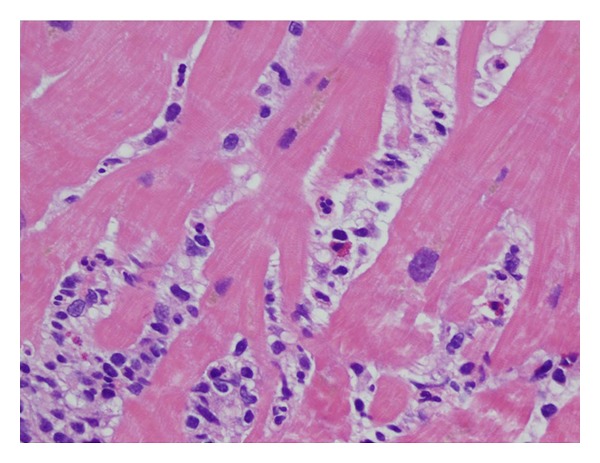
Right ventricular biopsy demonstrating interstitial inflammatory cell infiltrate in the myocardium, H&E stained with ×400 magnification. Most infiltrating cells are mononuclear cells, admixed with occasional eosinophils.

**Figure 5:**
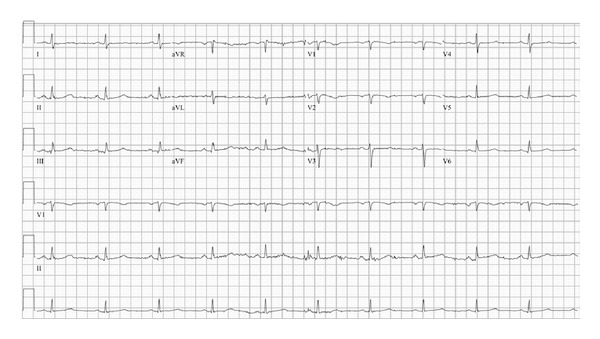
ECG after discharge shows sinus rhythm and no LBBB.

She was transferred to our hospital and was found to be in acute cardiogenic shock with acute pulmonary edema suggestive on chest plain films (Figure 2). She was started on inotropes, continuous Lasix infusion, was intubated, and had an IABP placed at bedside. Transthoracic echocardiogram showed LVEF <20% without significant valvular pathology or regurgitation with normal ventricular size, LVED 4.1 cm (Figure 3). Right heart catheterization was consistent with cardiogenic shock. Cardiac magnetic resonance imaging (MRI) was deferred due to the presence of cervical and lumbar orthopedic hardware. She received solumedrol 1 gram intravenous due to clinical suspicion for acute myocarditis. The LBBB and atrial fibrillation resolved after two hours of solumedrol. On hospital day (HD) 2 she remained on mechanical ventilation, continued inotropic support with dobutamine and milrinone, and continued afterload reduction with an IABP. An EMB showed aggressive myocarditis with predominant monocytes and neutrophils with rare eosinophils (Figure 4). She was continued on intravenous solumedrol 250 mg every six hours. IABP was weaned, but she developed ventricular bigeminy. This prompted administration of IVIG 40 g via intravenous bolus in attempt to prevent myocarditis progression. This management led to hemodynamic stability, normal cardiac electrical activity, improved organ perfusion, and pulmonary edema resolution. IVIG treatments were given daily and discontinued after four doses. Gradually diuretics were discontinued, inotropes were weaned, and IV steroids were transitioned to oral prednisone that was tapered over 5 days. Serum studies were negative for bacterial or viral antibodies that would suggest recent infection. These studies included serum or blood antigens for *Streptococcus pneumonia*, *Borrelia burgdorferi*, *Chlamydia pneumonia*, *C. trachomatis*, *C. psittaci*, legionella, *Trypanosoma cruzi*, *M. tuberculosis*, toxoplasma, hepatitis B/C, EBV, CMV, HIV1/2, coxsackie panel, adenovirus, mycoplasma, influenza A/B, RSV A/B, metapneumo virus, parainfluenza 1–3, and Parvovirus B19. On HD 7 she was extubated and weaned to room air. Repeat cardiac echocardiography showed a normal ejection fraction with complete resolution of severe biventricular failure (Figure 3) and chest X-ray confirmed resolution of pulmonary edema. On HD 10, she was discharged home without dyspnea or chest pain. Last echo showed LVEF 60% with normal ventricular size and EKG normalized (Figure 5).

## 3. Discussion

Myocarditis is a rare disease with a wide spectrum of clinical presentation, ranging from anginal chest pains to fulminant heart failure and death [1]. Diagnosis can be challenging and specific etiology elusive. Excluding postmortem exam, the gold standard diagnosis of myocarditis requires endomyocardial biopsy (EMB). Class I indications for biopsy only include those with acute severe heart failure requiring inotropic or mechanical support, symptomatic ventricular tachycardia, high degree heart block, or those that fail to respond to standard therapy [3]. For patients who do not meet these criteria for EMB, cardiac MRI is a valuable alternative diagnostic tool to distinguish between different patterns of pathology and help guide diagnosis [4]. In our patient, we deferred MRI because we could not exclude the presence of ferromagnetic orthopedic hardware. Determining etiology of myocarditis is important, as many primary immune processes improve with targeted therapies.

Treatment for acute fulminant myocarditis is primarily supportive following evidence-based recommendations [1]. The use of an intra-aortic balloon pump or other methods of left-ventricular mechanical support have been shown to improve outcomes [5]. Some clinicians use high dose steroids and IVIG to treat the underlying inflammatory pathology in myocarditis. However, immunologic based therapies have remained controversial for most types of myocarditis. While several studies have shown benefits of steroids and IVIG in myocarditis, randomized controlled trials have failed to show a benefit [1]. Patients with acute dilated cardiomyopathy who were treated with IVIG had similar LVEF 12 months after treatment compared to those given placebo [6]. However, most patients show significant improvement with supportive measures alone [7]. Also, there are many limitations inherent in available studies. The Dallas criteria may misdiagnose myocarditis and many current trials lack appropriate molecular biologic analysis of EMB to diagnose specific etiology [1]. In the context of these limitations, there is no clear support for immunosuppressive therapy in patients with known viral myocarditis. Evidence does support the use of immunosuppressive therapy in those with giant cell myocarditis, sarcoidosis, and primary autoimmune disease such as lupus [1, 8, 9]. It is often difficult to rapidly diagnose these etiologies. Thus, some clinicians initiate steroids with or without IVIG when patients clinically decompensate despite advanced cardiopulmonary support in an effort to treat cryptogenic primary immune processes. Although IVIG treatment is controversial and its use cannot be routinely recommended, it is possible that in select cases when myocarditis has been proven and there is no response to intensive supportive measures, IVIG may be considered.
